# A Rare Case of Ectopic Colonic Mucosa Presenting With Airway Compromise in a Neonate

**DOI:** 10.7759/cureus.27031

**Published:** 2022-07-19

**Authors:** Justin Hall, Fatima Z Aly, Julia Comer, Michael P Gebhard, Thomas Schrepfer

**Affiliations:** 1 Otolaryngology, Wake Forest School of Medicine, Winston-Salem, USA; 2 Department of Pathology, Immunology, and Laboratory Medicine, University of Florida College of Medicine, Gainesville, USA; 3 Otolaryngology, University of Illinois at Chicago, Chicago, USA; 4 Otolaryngology, University of Florida College of Medicine, Gainesville, USA

**Keywords:** oropharynx, congenital mass, neonatal respiratory distress, enteric duplication cyst, pediatric otolaryngology, pathology

## Abstract

This case report documents a rare and unique presentation of an oropharyngeal duplication cyst and subsequent neonatal airway management. A one-day-old premature female presented with postpartum respiratory distress requiring emergent intubation secondary to an oropharyngeal mass of the left tongue. After being stabilized and transferred to an academic center, imaging revealed a cystic lesion that was then marsupialized and drained by the otolaryngology team. Pathology demonstrated mature colonic tissue and was consistent with an enteric duplication cyst. This report highlights the importance of prenatal diagnosis and the potential of a lifesaving ex utero intrapartum treatment (EXIT) procedure.

## Introduction

Enteric duplication cysts can be found anywhere along the alimentary canal and are benign congenital heterotopic tissue with two layers of smooth muscle, gastrointestinal, or respiratory epithelium [1,2]. These cysts are rare entities and have a reported incidence of one per 4,500 births, with a slight male predominance [3]. Gastrointestinal tract duplication cysts usually present with gastric type epithelium and can occur anywhere from the oral cavity to the rectum, but they are most commonly found in the ileum, followed by the esophagus, colon, jejunum, stomach, and duodenum [1,2,4-6]. Their presence within the oral cavity is rare - only 0.3% of cases are seen in the tongue [4,7]. The majority of enteric duplications are discovered before the age of two years and usually require surgical resection for definitive treatment [7,8]. Clinical presentation may vary depending on the size and localization of the malformation. Symptomatic individuals present with difficulties in feeding, swallowing, and breathing [6,7]. Perinatal imaging is crucial for prenatal management, successful delivery, and surgical planning in neonates with an airway lesion. In this report, we present a case of a preterm neonate presenting with respiratory distress secondary to an oropharyngeal mass, thus requiring emergent intubation and transfer to a tertiary care facility. Imaging, drainage, and excision with marsupialization were performed, and an intramural colonic duplication cyst was found within the left aspect of the base of the tongue on pathological analysis. We discuss the accuracy of embryologic, histopathologic, and radiologic data, and emphasize the importance of prenatal diagnostics and the potential of a lifesaving ex utero intrapartum treatment (EXIT) procedure for airway management.

## Case presentation

A one-day-old female born preterm at 33 weeks and six days was transferred to our tertiary referral hospital for evaluation and treatment of an oropharyngeal mass causing airway obstruction. She had been born to a gravida 3 para 3 26-year-old African American female by spontaneous vaginal delivery after she had a premature rupture of membranes and a positive group B Streptococcus culture. Antenatal ultrasound had not been performed during the pregnancy. Surfactant had been administered at the outside hospital; Apgar scores at five and 10 minutes had been 4 and 6, respectively, and the patient had been noted to be in respiratory distress with poor respiratory effort. The patient weighed 2,170 grams. Positive pressure ventilation with a bag valve mask had been initiated and intubation had been attempted, which had led to the discovery of the oropharyngeal mass. A laryngeal mask airway had been immediately placed. The otolaryngology service had then performed direct laryngoscopy and intubation with a 3.0 endotracheal tube utilizing a Parsons laryngoscope. The patient had then been transferred to our facility for further management and care.

**Figure 1:**
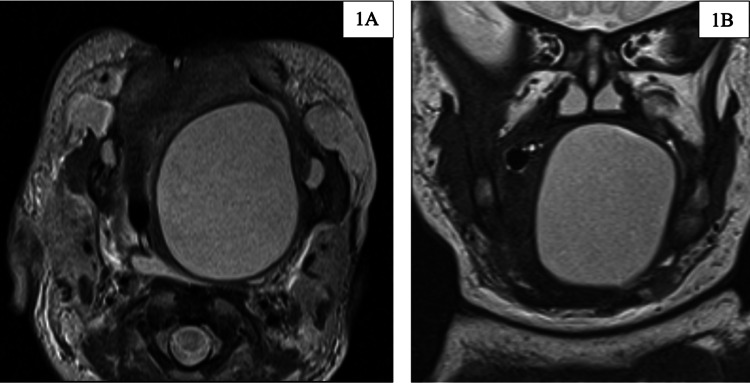
MRI of the oral cavity Axial (1A) and coronal (1B) T2 images of a large, well-circumscribed, non-enhancing cystic lesion in the left tongue base, measuring up to 3.6 cm MRI: magnetic resonance imaging

On arrival, the patient was noted to be hypoglycemic (29 mg/dL). An umbilical catheter was placed for resuscitation and a nasogastric tube was placed for enteral feeding. Ampicillin was given for sepsis prophylaxis. MRI of the oral cavity was performed on day two of life (Figures 1A, 1B).

**Figure 2:**
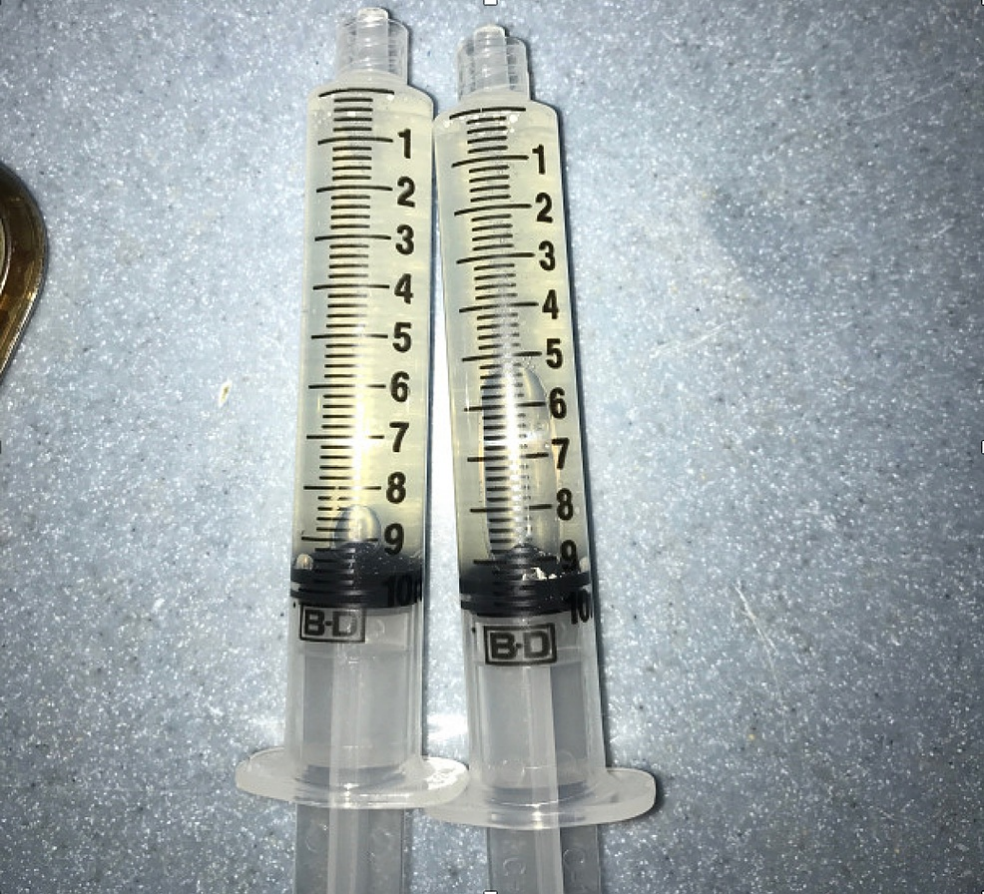
Cyst aspirate A total of 20-mL cloudy mucoid cystic content was aspirated and sent for cytology

**Figure 3:**
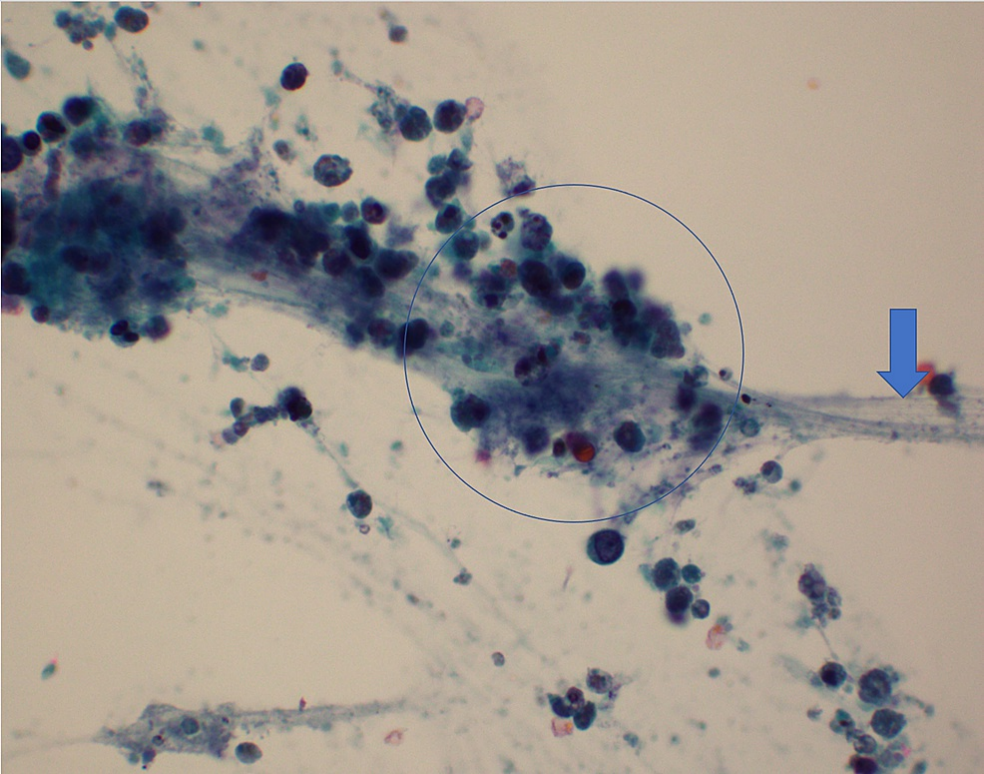
Cytology of cyst fluid showing degenerate epithelial cells (circled) in a background of mucin (arrow) (Papanicolau stain x600)

Based on the MRI, the radiologist described a large well-circumscribed non-enhancing cystic lesion in the left tongue base and oral cavity measuring up to approximately 3.6 cm. This was thought to represent a dermoid cyst or teratoma or, less likely, an epidermoid cyst or lymphangioma. On day two of the patient's life, the cyst was aspirated and drained at the bedside. A total of 20 mL of mildly cloudy, mucoid cystic contents were aspirated and sent for cytology (Figure 2). Cytologic examination of aspirate showed macrophages, blood, amorphous debris, rare bland non-specific cyst lining cells, and no evidence of malignancy (Figure 3).

**Figure 4:**
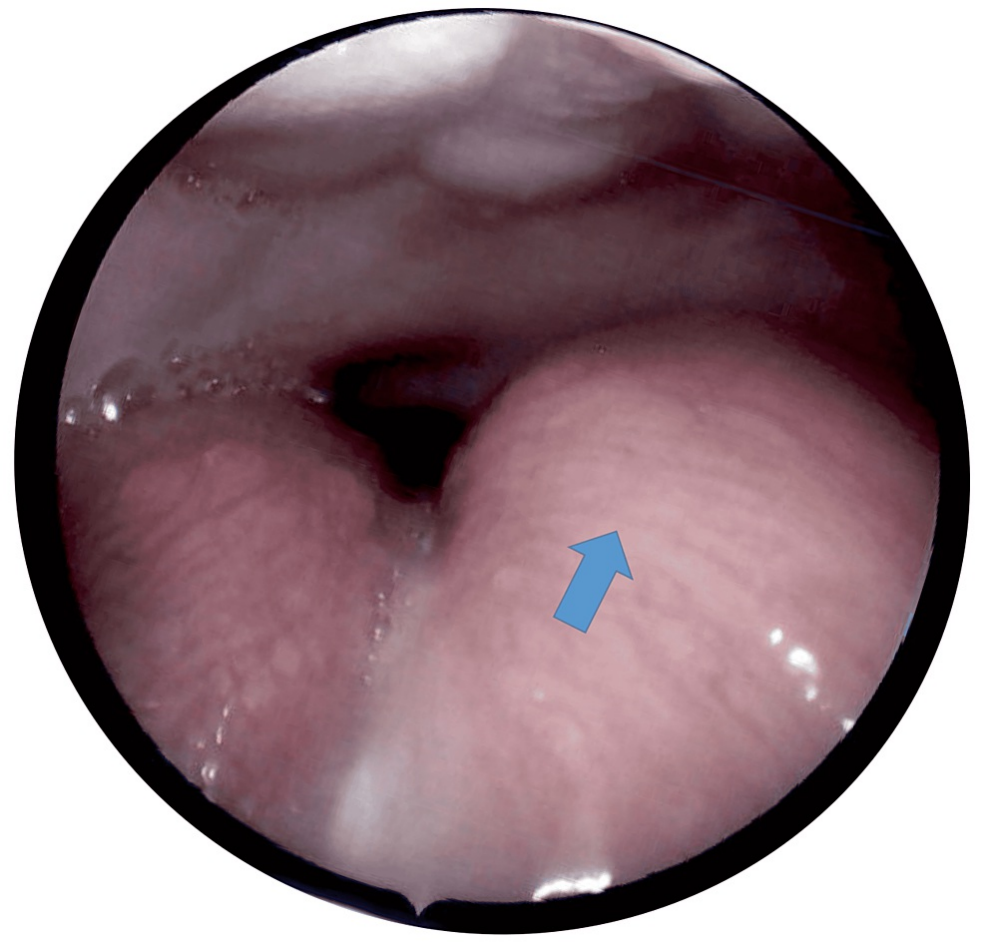
Preoperative photograph of the left tongue cyst (arrow) Hard palate (superior), normal tongue with tongue base papillae visible. Note that this photograph was taken several days after aspiration and drainage of the cyst but before the marsupialization

**Figure 5:**
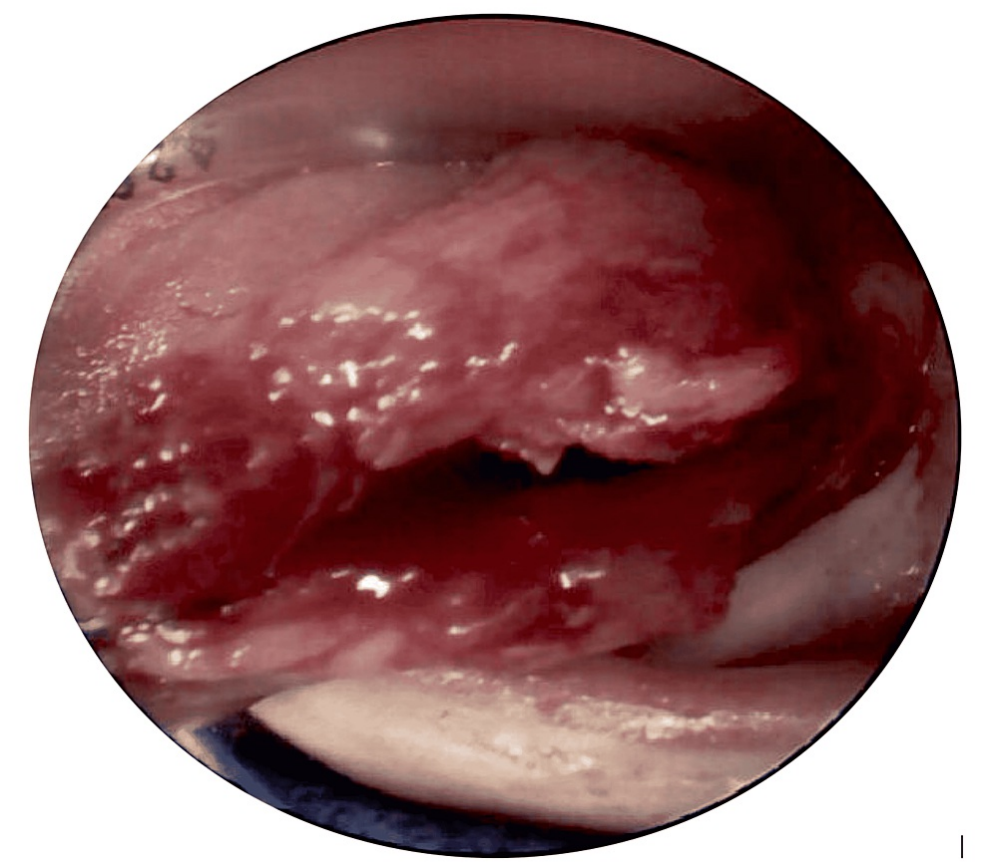
Intraoperative photograph of the marsupialization of the tongue cyst with the tongue being retracted out of the oral cavity The incision was made along the left lateral tongue. The lower lip (inferior) and endotracheal tube (superior, along the dorsal tongue) are also in view

Throughout the patient’s hospital stay, the only other complication observed was metabolic acidosis, which was managed with sodium acetate. On day eight of life, interventional radiology performed an image-guided Jackson-Pratt drain placement (using fluoroscopy and ultrasound) that removed an additional 6 mL of hemorrhagic fluid. After reviewing the test results available, the most likely working diagnosis was an oropharyngeal duplication cyst. Sclerotherapy was not recommended. On day 15 of life, the patient was extubated, and the nasogastric tube was removed. On day 16 of life, our pediatric otolaryngologist performed microlaryngoscopy, bronchoscopy, and excision biopsy with marsupialization of the tongue cyst (Figures 4, 5).

**Figure 6:**
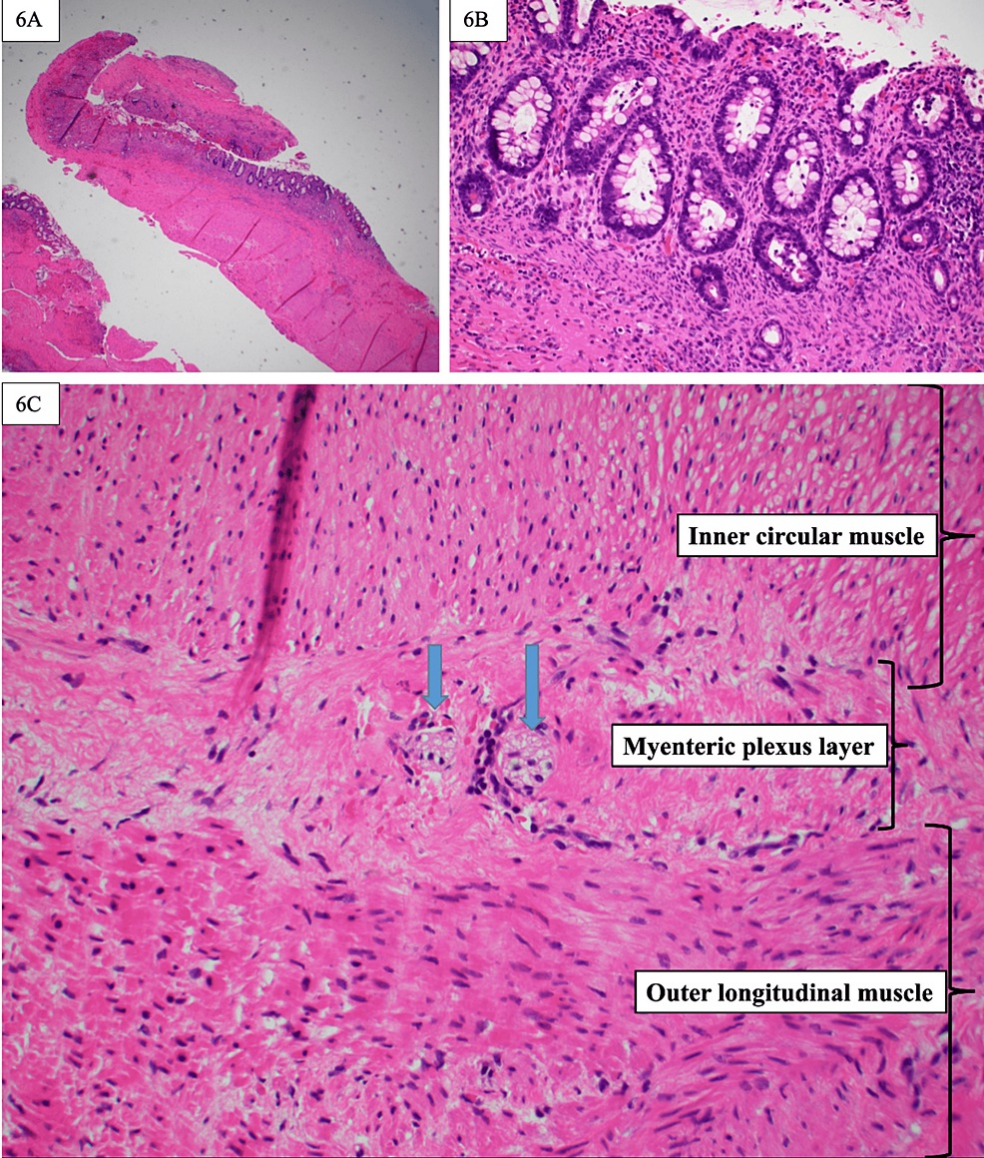
Hematoxylin and eosin stains of oropharyngeal cyst 6A) Cyst wall showing glandular epithelium and underlying thick wall (x10). 6B) Colonic type mucosa with goblet cells and crypts (x20). 6C) Ganglion cells (arrows) located between the inner circular and outer longitudinal layers of the muscularis propria (x40)

Attempts were made intraoperatively to separate the cyst wall from the surrounding tongue muscle, but it was noted to be integrated into the underlying muscle. Operative specimens were sent to pathology for histologic examination. The pathology report revealed fragments of the cyst wall composed of the colonic mucosa and the underlying enteric smooth muscle layers, with features most consistent with the diagnosis of an oropharyngeal duplication cyst (Figures 6A-6C).

The postoperative course was uncomplicated, and the patient was extubated on postoperative day one. Progression to full oral feeding was achieved during the patient’s hospital stay itself. On follow-up examination at two months of age, the patient had a well-healed base of the tongue with only a small area of a possible remnant on the left tongue. No further interventions were necessary as the patient was thriving, feeding orally, and her parents had no other concerns.

## Discussion

Enteric duplication cysts are usually detected prenatally or in the first year of life, with variable clinical significance, depending on size, location, and type of mucosal pattern. The nomenclature of enteric duplication cysts includes choristoma, heterotopic gastrointestinal cyst, and enterocystoma [9]. While the majority of those presenting in the oral cavity have gastric tissue, around 16% will have intestinal tissue [4]. The currently accepted theory behind their pathogenesis proposes that there is a disturbance in notochord development causing segments of the alimentary canal to be misplaced. This would also account for the association of enteric duplication cysts with other anomalies [10]. Other theories include small epithelial inclusions being trapped during primordial tissue fusion and the persistence of epithelial buds within the wall of the bowel [10].

The differential diagnosis for an oral tongue lesion in a neonate remains broad and includes teratoma, cystic hygroma, dermoid cyst, ranula, hemangioma, neuroblastoma, enteric duplication cyst, and thyroglossal duct cyst [11-12]. In an ideal setting, this differential can be determined in the prenatal period. The primary imaging technique in the antenatal period is ultrasound, but diagnosis of a tumor involving the oral cavity/oropharynx can be difficult. Prenatal ultrasound has low sensitivity unless the mass is significantly large or there are other abnormalities detected that require further investigation, i.e., polyhydramnios, or anatomic malformations [13]. Enteric duplication cyst ultrasound findings are usually difficult to identify when presenting in the bowel but will have a “double-wall or muscular rim” caused by inner hyperechoic mucosa and outer hypoechoic smooth muscle layer [14]. Antenatal ultrasound has been useful in the detection of intraoral cysts and some cases report the utility of Doppler ultrasound in detecting airway compromise by evaluation of amniotic flow [15]. Our patient had not undergone a prenatal ultrasound evaluation, which would have enabled preemptive management. One of the modalities for antenatal planning that has increasing use is fetal MRI. MRI characteristics of these cysts include hyperintensity on T2, short T1 inversion, and no enhancement after contrast [15]. In this case, the MRI ruled out both vascular and lymphatic malformation. Based on the presence of a mucosal lining seen on the MRI and during the drainage by interventional radiology, it was determined that sclerotherapy would not be ideal due to the risk of absorption of potentially toxic agents.

From this point onward, the case became a pathologic diagnosis. The pathological diagnostic criterion for enteric duplication cysts is the presence of a smooth muscle layer wall that contains epithelium derived from the alimentary canal and is located along the alimentary tract [16]. Perhaps the most interesting part of this case’s workup was the discrepancy between the radiological and pathological diagnoses.

A comparison of teratomas and enteric duplication cysts is insightful in this discussion, as both were considered potential diagnoses based on the initial MRI findings. Ultimately, the pathological analysis established the final diagnosis of enteric duplication cyst. Part of the difficulty in this diagnosis stemmed from the cyst’s location on the tongue. While the cyst was labeled as the base of the tongue, in actuality, there was not a clear distinction as a cyst residing solely at the base of the tongue. Possible involvement of the ectodermal tissue anterior to the circumvallate papillae could have favored the diagnosis of mature teratoma. Histologically, teratomas are more often seen as having tissue from all three germ cell layers and are composed of disorganized tissue. In comparison, enteric duplication cysts have highly organized tissue and are derived from the endodermal lining [5]. This case’s tissue was highly organized into an entire colonic layer, which supported the diagnosis of enteric duplication cyst. This case is particularly fascinating because of the complete demonstration of an organized colonic mucosal layer with underlying smooth muscle and ganglion cells (Figures 6A-6C).

In any congenital malformation, the initial management depends on the hemodynamic and respiratory status of the patient. In cases where respiratory compromise is noted, the first step in the management is establishing an airway. This can be particularly difficult, and in some cases, one may need to use fiberoptic laryngoscopy or tracheostomy if initial measures fail. Airway obstruction can present at birth, or weeks to months after delivery. If oral intubation is unattainable, being prepared for tracheostomy can be lifesaving for newborns as well [13]. Many of the documented congenital oral cysts present with respiratory compromise [17]. The mortality rate for children with base-of-tongue cysts is about 40% [18]. Therapeutic fine needle aspiration with drainage of the fluid has been found useful in cases to temporarily reduce airway obstruction [2]. One of the more recently described procedures for neonates suspected to have airway obstruction in the newborn period is the EXIT procedure.

The EXIT procedure involves maintaining uteroplacental blood flow via cesarean section delivery while attempting to immediately secure the airway. The goal is to achieve partial cesarean delivery of the head, neck, and torso with the advent of uterine-relaxing agents. Uteroplacental bypass time averages 24 minutes, which extends the amount of time able to perform lifesaving airway management [19]. The main risk of the EXIT procedure is increased maternal uterine bleeding. Survival rates for infants with this procedure have been around 90%, with mortality occurring in neonates with larger, more obstructive masses [19]. Had this patient’s mass been recognized on prenatal imaging, she could have been a potential candidate for a safer delivery with the EXIT procedure.

## Conclusions

We reported a rare case of colonic tissue within a base-of-tongue cyst presenting as airway obstruction. The final diagnosis was based on the correlation of clinical, radiological, and histological findings. We believe this case represents a variation of enteric duplication cyst presenting in a rare location in the head and neck that caused airway obstruction and immediate threat to the life of this newborn. Treatment of such cases should be multidisciplinary and coordinated with as much prenatal planning as possible prior to the delivery to ensure a safe, secure airway and definitive treatment of the obstructing mass.
